# Incorporation of Graphene Oxide Modified with Polyamide Curing Agent into the Epoxy–Zinc Composite Coating for Promoting Its Corrosion Resistance

**DOI:** 10.3390/polym15081873

**Published:** 2023-04-13

**Authors:** Shengjun He, Guangxiong Wei, Zhengnan Zhang, Lifeng Yang, Yuebin Lin, Longji Du, Xusheng Du

**Affiliations:** 1 China Railway 11th Bureau Group Second Engineering Co., Ltd., Wuhan 430061, China; 2 CRCC Harbour & Channel Engineering Bureau Group Co., Ltd., Zhuhai 519070, China; 3 Zhuhai Communication Group, Zhuhai 519000, China; 4 The Key Laboratory of Urban Security and Disaster Engineering of Ministry of Education, Beijing University of Technology, Pingleyuan Road 100, Beijing 100124, China; 5 Highway Bridges National Engineering Research Center, Beijing 100088, China; 6 Institute of Advanced Wear & Corrosion Resistant and Functional Materials, Jinan University, Guangzhou 510632, China

**Keywords:** graphene oxide, intercalation, carbon steel, epoxy-zinc coating, corrosion resistance, surface modification

## Abstract

To promote the anticorrosion performance of epoxy/zinc (EP/Zn) coating, graphene oxide (GO) was directly incorporated into dual-component paint. Interestingly, it was found that the method of incorporating GO during the fabrication of the composite paints strongly influenced their performance. The samples were characterized by Fourier transform infrared spectroscopy (FT-IR), X-ray diffraction (XRD), and Raman spectroscopy. The results indicated that GO could be intercalated and modified with the polyamide curing agent while preparing component B of the paint, for which the interlayer spacing of the resulting polyamide modified GO (PGO) increased, and its dispersion in organic solvent was improved. The corrosion resistance of the coatings was studied through potentiodynamic polarization testing, electrochemical impedance spectroscopy (EIS), and immersion testing. Among the three types of as-prepared coatings, i.e., neat EP/Zn coating, GO modified EP/Zn coating (GO/EP/Zn), and PGO-modified EP/Zn coating (PGO/EP/Zn), the order of the corrosion resistance of the coatings was PGO/EP/Zn > GO/EP/Zn > neat EP/Zn. This work demonstrates that although the in situ modification of GO with a curing agent is a simple method, it evidently promotes the shielding effect of the coating and enhances its corrosion resistance.

## 1. Introduction

Coatings are often unitized to protect metals from corrosion [1,2,3,4]. Among the various coatings, Zn-rich polymer composite paint is a widely applied type of coating due to the effect of the Zn particles, which work as anodes to provide cathodic protection for metal substrates [1,5,6,7,8]. In most of the commercial products, a heavy loading of Zn particles, up to 80% of the content, has to be used to achieve satisfying anticorrosion performance [8]. However, this high Zn content in the coating has its own disadvantages, resulting in poor mechanical coating properties, the large consumption of Zn, and the high economic cost of the paint. In order to make the best use of Zn particles in the polymer matrix in the coatings and improve their corrosion resistance, additional nanofillers have been incorporated into these coatings. For instances, various particles, such as carbon nanotubes [9], alumina [10], metal sulfides [11], and clay [7], have been added into Zn-containing polymer composite coatings.

Two-dimensional (2D) nanocarbon materials, including carbon or graphene sheets, have been directly deposited onto metal substrates as protective coatings via CVD or flame deposition [12,13,14]. They exhibit excellent corrosive and scratch resistance. The addition of graphene to polymer composite coatings is another efficient way to make use of these 2D nanocarbon materials in coating systems, where their barrier property can be fully exploited [15,16,17,18,19,20,21,22]. For Zn/polymer composite coating systems, 2D carbon sheets also promote the formation of electron conduction paths between Zn particles, thereby enhancing the cathodic protection of the coating.

Given its high aspect ratio and chemical inertness, graphene is usually modified before introducing it into resin matrices with the aim of enhancing its interactions with the matrix and/or improving its dispersibility in the coating [23,24,25,26,27,28,29,30,31]. As an oxidation product of graphene, graphene oxide (GO) can be simply defined as graphene with many organic functional groups on its surface, which can be directly prepared from natural graphite with the well-known Hummers’ method [32,33]. Due to the many functional groups on its surface, GO has good dispersion in both aqueous solutions and water-soluble polymer matrices. Extensive studies have been devoted to modifying GO to improve its dispersion in organic solvents and coatings [26,27,28,29,30,31] as well as to improve its corrosion resistance via the loading of corrosive inhibitors on its surface [34,35]. However, the addition of the modification step increases the complexity and cost of the fabrication of paints.

In this study, GO modified with the polyamide curing agent was incorporated into dual-component epoxy/Zn paints. With the in situ preintercalation of GO with polyamide in the curing agent component of the paints, the interlayer of the modified GO was increased, and its dispersion in the organic solvent was improved. The physical and chemical properties of the polyamide modified GO were comprehensively characterized. The effects of the incorporation of GO and its addition order on the anticorrosion performance of an epoxy/Zn composite coating was investigated with various electrochemical techniques.

## 2. Experimental Materials and Instruments

### 2.1. Experimental Materials

Graphene oxide (GO) was purchased from Suzhou Tanfeng Technology Co., Ltd., Suzhou, China. Epoxy resin (E44) was ordered from Xiya Reagent, Linyi, China. Zylene, n-butanol, and polyamide 651 (PA-651) were purchased from McLean Group, Shanghai, China. Carbon steel plates (Q235, size: 30 mm × 40 mm) and Zn flake powder (size: 5–15 µm) were obtained from Fuquan Metal Co., Ltd., Quanzhou, China and Xiamen Empire New Material Technology Co., Ltd., Xiamen, China.

### 2.2. Preparation of the PA-651-Modified GO (PGO)

Specifically, PGO could be obtained via the direct interaction between GO and PA-651. Similar to the preparation of component B during the fabrication of the dual-component epoxy paint, GO (0.03 g) was added to 1.5 g of PA-651 and mixed via magnetically stirring at 50 °C for 2 h. In order to extract the modified GO for characterizing its structure, the mixture was filtrated and then successively washed with acetone and deionized water. The product was then obtained by freeze-drying and named PGO.

### 2.3. Preparation of the Coating Samples

Before applying the coating, the Q235 steel sheets were successively washed with alcohol and then deionized water. After being sandblasted, the samples were ultrasonically cleaned in acetone. Two types of coatings were produced by adding GO in different orders into dual-component epoxy paint, i.e., component A (epoxy resin and Zn powder) and component B (PA-651, a curing agent). For the fabrication of the normal GO-incorporated coating, GO powder was added to component A and magnetically stirred at room temperature for 2 h. It was then mixed with component B to prepare the GO/EP/Zn coating. In contrast, the PGO/EP/Zn paint was prepared by firstly mixing GO with PA-651 at 50 °C and then magnetically stirring for 2 h to produce component B, which was subsequently mixed with component A. In all the coatings, organic solvent (xylene: n-butanol = 7:3) was utilized to dilute component A, and its mass fraction was 20 wt.% of the whole paint.

Finally, the coating was applied to the surface of the sandblasted Q235 steel sheets with a steel rod applicator and cured in an oven at 60 °C for 24 h. The thickness of the dry coating was 150 ± 10 µm. In addition, neat epoxy/Zn coating was prepared as the reference sample using the same method mentioned above.

### 2.4. Characterization Methods

The chemical functional groups of the sample were characterized with a Fourier transform infrared spectrometer (Thermo Scientific Nicolet iS50) over a scan range of 400 to 4000 cm^−1^. Both GO and PGO samples were characterized with Raman (Invia Renishaw Raman) and X-ray photoelectron spectroscopy (XPS; K-Alpha+, Thermo Scientific, East Grinstead, UK). An X-ray diffraction analyzer (XRD, Rigaku UItima IV, Nagano, Japan) was used to characterize the crystalline structure of the sample. Thermogravimetric analysis (TGA) and differential scanning calorimetry (DSC) tests of the samples were performed under N_2_ protection using a TGA/DSC 3+ thermogravimetric analyzer (Mettler Toledo International Co., Ltd., Zürich, Switzerland) at a heating rate of 5 °C min^−1^.

The electrochemical corrosion of the coatings was measured on a CHI 760e electrochemical workstation. A three-electrode system was constructed with a saturated calomel electrode (SCE) as the reference electrode, a platinum plate electrode as the auxiliary electrode, and 3.5 wt.% NaCl as the electrolyte solution. The potentiodynamic polarization test was carried out at a scan rate of 10 mV s^−1^, and the electrochemical impedance spectroscopy (EIS) tests were performed in a frequency range 10 kHz to 0.1 Hz under an amplitude voltage of 5 mV at room temperature.

## 3. Results and Discussion

### 3.1. Physical Chemical Structure of GO and PGO

**Figure 1:**
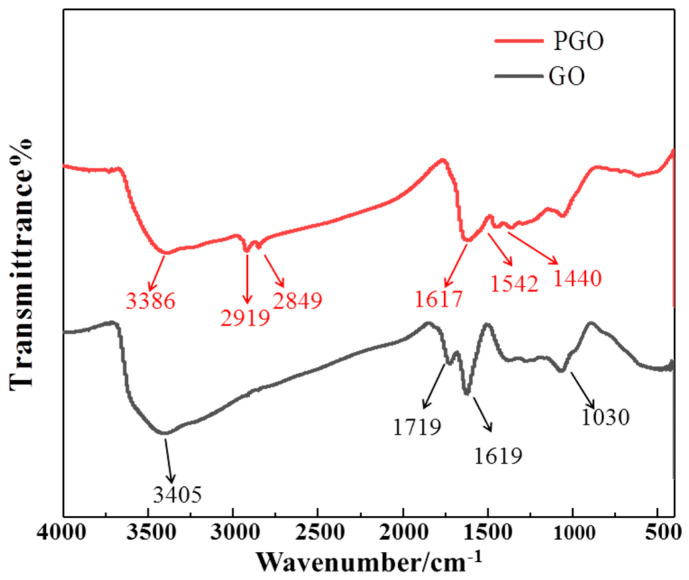
FTIR spectrum of PGO and GO.

As shown in Figure 1, the characteristic peaks of GO appeared at 1719 cm^−1^ (C=O carboxyl stretching vibration), 1619 cm^−1^ (C=C in aromatic ring), and 1066 cm^−1^ (C–O–C in epoxide), which is similar to the peaks reported in previous studies [32,35,36]. In addition, the strong and wide peak at 3000–3500 cm^−1^ could be ascribed to the hydroxyl groups. Two new peaks appeared at 2919 cm^−1^ and 2849 cm^−1^ in the FT-IR spectrum of PGO, and they originated from the stretching vibration of the alkyl group in polyamide 651, indicating the presence of a macromolecule chain in PGO (Figure 1). Moreover, a new peak at 1542 cm^−1^ (N–H stretching vibration), combined with that of the C=C in the aromatic ring (1617 cm^−1^), appeared along with a weakened peak at 1440 cm^−1^, corresponding to O=C–NH in the spectrum of PGO, identifying the presence of O=C–NH bands due to the intercalation and modification owing to PA-651. Furthermore, the weakened peak for O=C almost combined with the strong peak for the C=C bands, confirming the partial reduction of GO in the PGO product.

**Figure 2:**
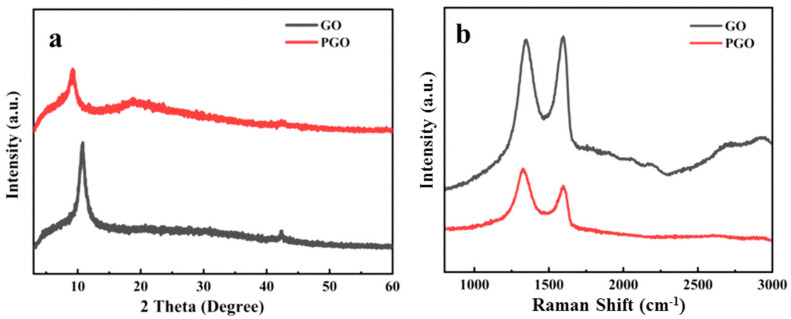
XRD pattern (**a**) and Raman spectra (**b**) of GO and PGO.

Figure 2a shows the XRD characterization results of GO and PGO. Due to the abundant oxygen-containing functional groups, the diffraction peak of GO is located at 2θ = 10.8°, which belongs to the (002) of GO, suggesting an interlayer spacing of 0.81 nm. After treatment with the curing agent, the diffraction peak of GO moved to a lower 2θ position and appeared at 2θ = 9.2°, which means that the interlayer spacing of PGO was about 0.96 nm. This indicates the increase in the interlayer spacing of GO, confirming the intercalation of PA 651 and the presence of functional groups in PGO.

Similar to other research on GO based carbon materials [2,3,4], two strong bands could be observed in the Raman spectra of both GO and PGO (Figure 2b). After the modification of GO with the polyamide, the D (1349 cm^−1^) and G bands (1597 cm^−1^) moved to 1329 cm^−1^ and 1596 cm^−1^, respectively, which indicated a certain charge transfer between the polyamide and GO. The intensity ratio between the D band and G band (I_D_/I_G_) is generally used to evaluate the quality of the carbon structure in various products (graphitization or defects). Due to the increase in isolated sp^2^ domains in the PA-651-modified GO, the intensity of the D band of PGO increased relative to that of pristine GO, resulting in an increase in the I_D_/I_G_ from 0.98 for GO to 1.25, which demonstrated the successful reaction and modification of GO with PA-651 macromolecules.

**Figure 3:**
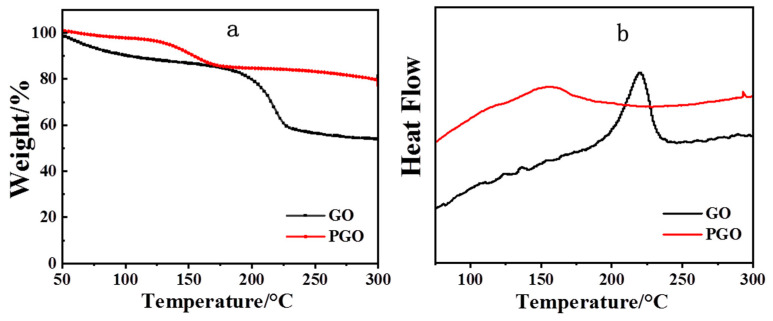
TGA(**a**) and DSC (**b**) curves of GO and PGO.

Figure 3 shows the TGA and DSC curves of the GO samples before and after the treatment with PA-651. GO displayed a major weight loss around 200 °C (Figure 3a), which we attributed to the removal of the oxygen-containing functional groups, while the weight loss below 100 °C could be ascribed to the removal of the physically adsorbed water in the hydrophilic sample [36]. In the TGA curve of PGO, the main weight loss started from 130 °C, which is much lower than that of GO. This weight loss could have been due to the decomposition of PA-651 and confirmed the successful modification of GO with the molecules. The DSC of GO (Figure 3b) showed one strong exothermic peak around 220 °C, which was caused by the decomposition of the organic-oxygen-containing groups on the GO sheets [36,37]. For PGO, such a strong exothermic peak was absent, and the weak peak that appeared around 155 °C likely originated from the decomposition of the PA-651 macromolecules grafted onto the GO sheets. These results agree well with those of TGA.

**Figure 4:**
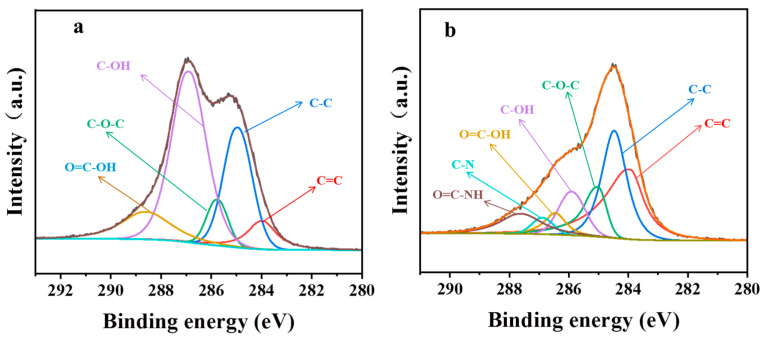
C1s spectrum of (**a**) GO and (**b**) PGO.

**Figure 5:**
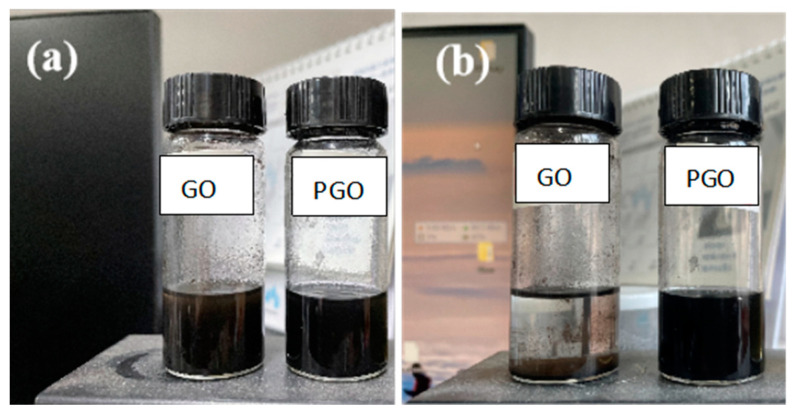
Digital photos of dispersion of GO and PGO in n-butanol: (**a**) just after ultrasonic irradiation and (**b**) after 15 min of standing.

The characteristic peaks in the C ls spectrum of GO according to XPS were related to C=C, C-C, C-OH, -C-O-C-, and COOH, (Figure 4a). The atomic ratio of O/C of PGO was 0.6, which is much less than that of GO (~1.3), confirming the large loss of oxygen-containing functional groups after the treatment with the curing agent. As shown in Figure 4b, two new peaks at 285.9 and 287.6 eV were detected in the C1s spectrum of PGO. They were related to C-N and HN-C=O in the PA-651 molecules grafted onto the carbon sheets, respectively. The atomic ratio of N/C of PGO was measured as 0.16. These experimental results confirmed that the polyamide macromolecules were grafted onto the surface of the GO. Additionally, the integral area ratio between the C=C and C-C peaks increased from 0.3 for GO to 1.2 for PGO, implying the occurrence of certain re-aromatization of GO in the PGO product. Furthermore, the advantage of first adding GO to PA-651 during the preparation of epoxy composite paints was demonstrated by its improved dispersion stability. As shown in Figure 5, the dispersion of PGO in n-butanol remained uniformly dark after 15 min of standing, in contrast with the almost complete precipitation of GO in the same solvent.

### 3.2. Corrosion Resistance of the Epoxy Coatings

#### 3.2.1. Corrosion Resistance of GO/EP/Zn Coatings

**Figure 6:**
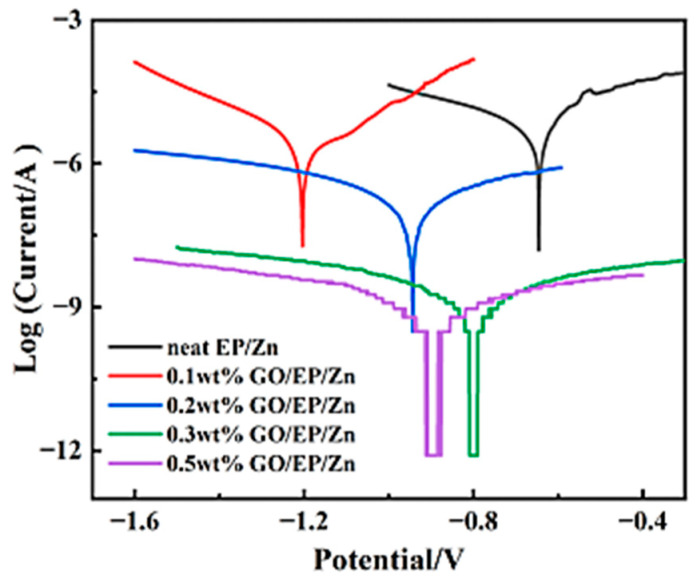
Tafel curve of the coating incorporated with different contents of GO in 3.5 wt.% NaCl solution.

The corrosion resistance performance of the EP/Zn coating was characterized with potentiodynamic polarization tests. Figure 6 shows Tafel plots of both neat EP/Zn and GO/EP/Zn coatings in 3.5 wt.% NaCl solution. It can be seen that with the increase in GO content from 0.1 to 0.3 wt.%, the polarization curves of the coating shifted downward along with decreasing corrosion current density (Icorr). The Tafel test results, including the Icorr and corrosion potential (Ecorr), are shown in Table 1. The Icorr of neat EP/Zn coating was close to that for Zn-rich epoxy coating reported in literature [6].

A lower content of GO (0.1 wt.%) was unfavorable and even deteriorated the corrosion inhibition performance of the coating, as its Icorr was larger than that of the neat EP/Zn coating. However, the Icorr of GO/EP/Zn coating decreased with the increasing GO content (Table 1). When the GO content was 0.3 wt.%, the Icorr of GO/EP/Zn was only 9.3 × 10^−10^ A cm^−2^, which indicated a nearly four orders of magnitude improvement in the corrosion resistance over that neat EP/Zn coating (6.1 × 10^−6^ A cm^−2^). However, when the GO content was further increased to 0.5 wt.%, its Icorr changed little (Table 1). Taking into account the cost of anticorrosion coatings, EP/Zn composite with 0.3 wt.% GO was determined to be the optimal coating for the surface protection of carbon steel and is called GO/EP/Zn in the following sections.

#### 3.2.2. Corrosion Resistance of PGO/EP/Zn Coating

**Figure 7:**
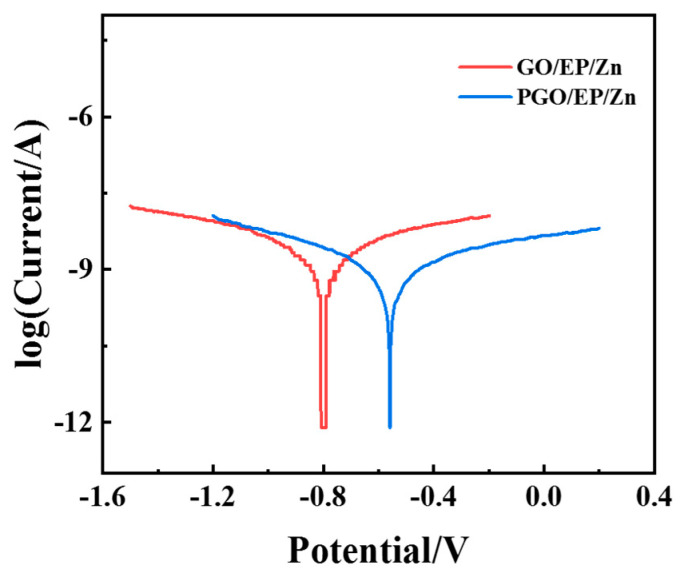
Tafel curves of EP/Zn coatings modified with GO and PGO in 3.5 wt.% NaCl solution.

Interestingly, we found that the corrosion resistance of the GO-modified EP/Zn coating could be further reduced by simply changing the order of the addition of GO during the fabrication of the epoxy composite paints. Different from adding GO first in component A when manufacturing GO/EP/Zn paints, GO was added to component B (which acted as the curing agent in the epoxy system) when preparing PGO/EP/Zn paint. With this technique, the optimized GO content (0.3 wt.%) was adopted, and the as-prepared coating was named PGO/EP/Zn. Its corrosion resistance performance was also characterized with potentiodynamic polarization tests. As shown in Figure 7, its Tafel curve shifted in the positive direction along with decreasing corrosion current density in comparison with that of GO/EP/Zn. Its Icorr was 4.9 × 10^−10^ A cm^−2^, which was nearly half that of GO/EP/Zn, as shown in Table 2. The enhanced corrosion resistance could be attributed to the different reaction environments for GO for the different addition orders. During the fabrication process of PGO/EP/Zn, the plentiful amine groups in the polyamide macromolecules made component B a highly reactive solvent for GO, which contained organic functional groups, such as carboxyl, hydroxyl, and epoxy groups. With the pretreatment and mixing with component B, GO was modified by PA-651 to form PGO in component B, resulting in an increased interlayer spacing and improved dispersion stability, thus allowing the exploitation of its barrier properties to prevent corrosion in the coating. In contrast, the active groups in the epoxy oligomer in component A are not chemical active for GO, and the resultant GO/EP/Zn fabricated with this addition order exhibited inferior corrosion resistance.

**Figure 8:**
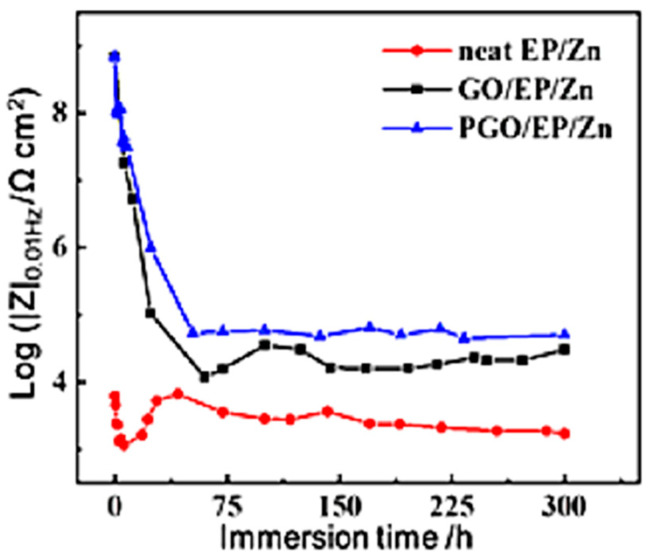
The dependence of the impedance for neat EP/Zn, GO/EP/Zn, and PGO/EP/Zn coatings on immersion time in 3.5 wt.% NaCl solution.

**Figure 9:**
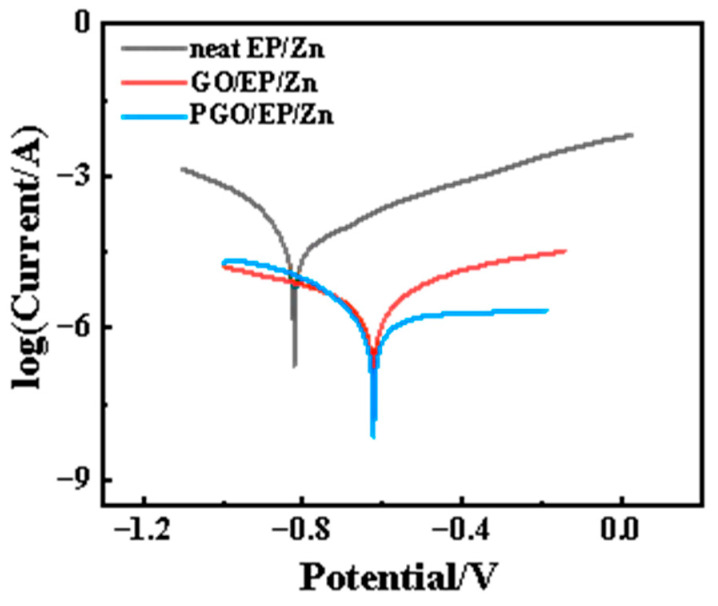
Tafel curve of neat EP/Zn, GO/EP/Zn, and PGO/EP/Zn samples after 300 h of immersion in 3.5 wt.% NaCl solution.

An immersion test was carried out to evaluate the long-term corrosion resistance performance of the three types of coatings. The impedance modulus at the lowest frequency (|Z|_0.01Hz_) in the Bode diagram of the coating is usually utilized as an important factor to identify the evolution of surface protection performance. As shown in Figure 8, the |Z|_0.01Hz_ of all the three samples substantially decreased with increasing immersion time within the first tens of hours of immersion, which was due to the gradual infiltration of the corrosive species from the 3.5 wt.% NaCl electrolyte solution into the epoxy composite coating, similar to the results of other work on Zn-rich epoxy coating [22]. It was noted that the |Z|_0.01Hz_ of neat EP/Zn decreased much faster than that of the GO/EP/Zn and PGO/EP/Zn samples, indicating the strong barrier property of GO and PGO sheets in the epoxy composite coating. In fact, the |Z|_0.01Hz_ of neat EP/Zn was much less than those of both the GO/EP/Zn and PGO/EP/Zn samples during the whole immersion test, confirming the positive effect of the 2D GO and PGO sheet on the promotion of the corrosion resistance of the EP/Zn coating. After 2 days of immersion, the |Z|_0.01Hz_ of all the three samples reached a relative stable state and insignificantly varied with increasing immersion time. Evidently, the stable part of the |Z|_0.01Hz_ curve of PGO/EP/Zn was higher than that of GO/EP/Zn, indicating the superior corrosion resistance of PGO/EP/Zn compared with that of GO/EP/Zn. Moreover, after 300 h of immersion, its Tafel curve remained lower than those of both neat EP/Zn and GO/EP/Zn, as shown in Figure 9. Its Icorr was 1.2 × 10^−6^ A cm^−2^, which was nearly half that of GO/EP/Zn (4.9 × 10^−5^ A cm^−2^) and was one order magnitude less than that of neat EP/Zn (Table 3).

**Figure 10:**
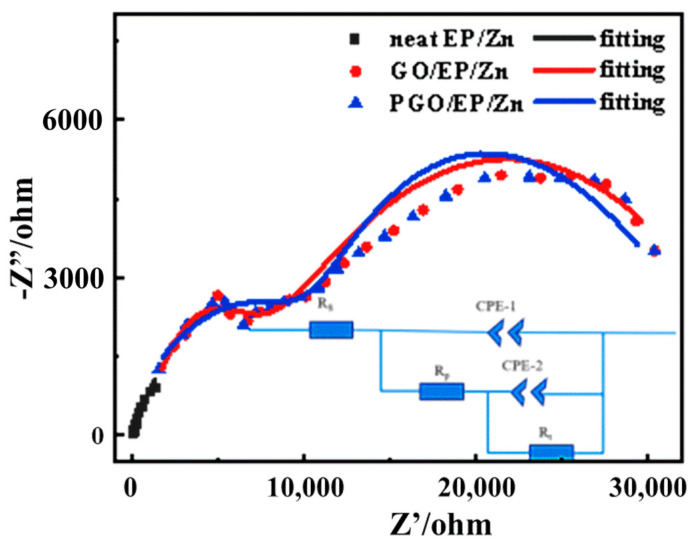
Nyquist diagrams of neat EP/Zn, GO/EP/Zn, and PGO/EP/Zn samples after 300 h of immersion in 3.5 wt.% NaCl solution and the fitted data based on the inset equivalent circuit.

In order to reveal the corrosion mechanism of the GO-incorporated EP/Zn coatings, Nyquist diagrams measured for the different coatings after 300 h of immersion in 3.5 wt.% NaCl solution were obtained, as shown in Figure 10. They were fitted and analyzed by the equivalent circuit, which is inset in Figure 10. In the equivalent circuit diagram, Rs refers to the solution resistance; CPE-1 and Rp represent the constant phase element value of the coating capacitor and coating pore resistance, respectively; CPE-2 and Rt represent the constant phase element and charge transfer resistance of metal-substrate double-layer capacitor, respectively [6,38]. The fitting component parameters for the EIS of GO/EP/Zn and PGO/EP/Zn in Figure 10 are shown in Table 4. As an important parameter of the fitted data in EIS, Rt is usually used to evaluate the corrosion resistance performance of a coating, where a high Rt value generally implies a low-speed electrochemical corrosion reaction. With the incorporation of GO, it was found that the Rt of both the GO/EP/Zn and PGO/EP/Zn coatings increased to varying degrees. We found that the Rt value of PGO/EP/Zn (64,520 Ω·cm^−2^) was more than twice that of GO/EP/Zn (31,830 Ω·cm^−2^), which agreed with the aforementioned Tafel results (Figure 9). This meant that the corrosion resistance of the EP/Zn coating was strongly promoted by the incorporation of 0.3 wt.% GO. In addition, a positive correlation exists between the Rp value and the physical barrier properties of a coating [1,6,38]. As shown in Table 4, the Rp value of PGO/EP/Zn was 13,115 Ω·cm^−2^, which was considerably more than that of GO/EP/Zn (5386 Ω·cm^−2^). This could be due to the grafting of PA 651 onto PGO, which led to its enhanced dispersibility in the coating and improved interaction with the matrix, therefore favoring the filling of micropores in the epoxy matrix and hindering the permeation of corrosives from the electrolyte solution.

**Figure 11:**
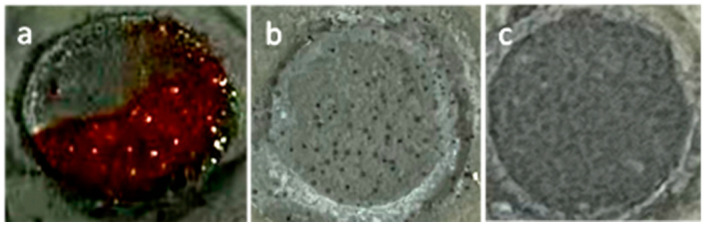
Visual observations of the different coating samples exposed to 3.5 wt.% NaCl solution for 300 h of immersion: (**a**) neat EP/Zn, (**b**) GO/EP/Zn, and (**c**) PGO/EP/Zn samples. The diameter of the circle in the image is 1.1 cm.

The surface morphologies of the different coatings after 300 h of immersion (the circle area) to 3.5 wt.% NaCl solution are shown in Figure 11. After the long-term immersion test, the neat EP/Zn coating displayed a surface morphology different from that of the GO-incorporated epoxy composite coatings. It was seen that more than half of its exposed surface was covered with red corrosion products. The red materials were likely ferric compounds, which originated from the corrosion of the steel substrate. However, such red materials were totally absent on the surface of the GO/EP/Zn and PGO/EP/Zn coating samples, confirming the greatly enhanced corrosion resistance of the GO-incorporated epoxy composite coatings. For the GO/EP/Zn coating, some blistering appeared on its exposed surface, which was in contrast with that of the featureless surface of the PGO/EP/Zn coating. This finding also demonstrated the superior corrosion resistance of the PGO/EP/Zn coatings.

## 4. Conclusions

In this study, GO was incorporated into epoxy/Zn composite paint to improve the corrosion resistance of the composite coating; the effect of the method of incorporating GO into the paint on this performance was studied as well. The main conclusions were drawn as follows:

1. The corrosion resistance of epoxy/Zn composite paint was significantly improved by directly incorporating GO into the coating, and the optimized GO content was 0.3 wt.%.
2. GO was successfully modified with PA-651 to form PGO in component B of the paint, resulting in increased interlayer spacing, higher thermal stability, as well as improved dispersion stability in the epoxy solvent.
3. Due to the polyamide modification and the physical shielding of GO nanosheets, the PGO-incorporated EP/Zn coatings displayed a lower corrosion current density and a larger impedance modulus than the GO/EP/Zn coatings, when GO was added into component A of the epoxy composite paint.
4. The modification of GO with the curing agent helped with filling the micropores in the epoxy coating, increasing the coating pore resistance and charge transfer resistance during the corrosion process.

## Figures and Tables

**Table 1:** Icorr and Ecorr of GO/EP/Zn coating with different GO contents in 3.5 wt.% NaCl solution.

Sample	Icorr (A·cm^−2^)	Ecorr (V)
Neat EP/Zn	6.1 × 10^−6^	−0.65
0.1 wt.% GO	3.3 × 10^−6^	−1.21
0.2 wt.% GO	1.0 × 10^−7^	−0.94
0.3 wt.% GO	9.3 × 10^−10^	−0.80
0.5 wt.% GO	8.8 × 10^−10^	−0.90

**Table 2:** Icorr and Ecorr of neat EP/Zn, GO/EP/Zn and PGO/EP/Zn coating with different GO contents in 3.5 wt.% NaCl solution.

Sample	Icorr (A·cm^−2^)	Ecorr (V)
neat EP/Zn	6.1 × 10^−6^	−0.65
GO/EP/Zn	9.3 × 10^−10^	−0.80
PGO/EP/Zn	4.9 × 10^−10^	−0.56

**Table 3:** Icorr and Ecorr of neat EP/Zn, GO/EP/Zn and PGO/EP/Zn coatings after 300 h of immersion in 3.5 wt.% NaCl solution.

Sample	Icorr (A·cm^−2^)	Ecorr (V)
neat EP/Zn	4.9 × 10^−5^	−0.82
GO/EP/Zn	2.2 × 10^−6^	−0.63
PGO/EP/Zn	1.2 × 10^−6^	−0.62

**Table 4:** Electrochemical parameters extracted from the EIS results for the three coating samples.

Sample	CPE1 (S·cm^−2^·s^n^)	Rp (Ω·cm^−2^)	CPE2 (S·cm^−2^·s^n^)	Rt (Ω·cm^−2^)
Neat EP/Zn	6.84 × 10^−4^	-	-	8370
GO	1.43 × 10^−7^	5386	1.43 × 10^−5^	31,830
PGO	1.64 × 10^−7^	13,115	1.30 × 10^−5^	64,520

## Data Availability

Not applicable.
